# Intersectional inequalities in COVID-19 hospitalisation and mortality: A nationwide MAIHDA study of Swedish adults across four pandemic waves (2020–2022)

**DOI:** 10.1016/j.ssmph.2026.101968

**Published:** 2026-09-19

**Authors:** Jesper Löve, Gunilla Priebe, Bo Burström, Ailiana Santosa, Nawi Ng

**Affiliations:** a School of Public Health and Community Medicine, Sahlgrenska Academy, University of Gothenburg, Box 453, Gothenburg, SE-405 30, Sweden; b Department of Global Public Health, Karolinska Institutet, Stockholm, SE-171 77, Sweden

## Abstract

**Background:**

COVID-19 has been described as a syndemic shaped by interactions between viral exposure, co-morbidities, and social inequalities. However, much of the literature relies on single-axis analyses and restricted time horizons, obscuring how health inequalities shifted across pandemic phases. Intersectionality theory conceptualises how social positions intersect within systems of power to produce context-dependent inequalities. Nationwide intersectional evidence on health inequalities across the full pandemic remains scarce.

**Methods:**

Drawing on intersectionality methodology, we conducted a nationwide register-based cohort study of all Swedish adults (≥18 years) from March 2020 to March 2022. Intersectional strata combined sex, income, education, and birthplace income classification. COVID-19 hospitalisation and mortality were analysed separately for younger (18–69 years) and older (≥70 years) adults across four waves using multilevel models (MAIHDA) capturing between- and within-stratum heterogeneity.

**Results:**

Sweden recorded 67,997 COVID-19 hospitalisations and 15,895 deaths. Single-axis analyses indicated higher risks among men, those with lower education, and migrants from lower-income countries. Intersectional analyses revealed substantial heterogeneity and temporal variation. Among younger adults, intersectional strata accounted for up to 25% of variation in mortality. Among LIC-born men aged ≥70, wave-1 mortality was nearly fourfold higher in the low-income/low-education stratum that in the high-income/high-education stratum. Among older high-income men, hospitalisation disparities between LMIC-born and Swedish-born strata narrowed substantially across educational levels, with only small differences remaining by wave 4.

**Conclusions:**

Inequalities in severe COVID-19 outcomes were intersectional and temporally dynamic. The findings highlight the importance of moving beyond static, single-axis approaches to understand evolving health inequalities and inform equity-oriented pandemic preparedness and response.

## Highlights

• Nationwide MAIHDA assessed inequalities across four COVID-19 waves.
• Intersectional strata explained substantial mortality variation in younger adults.
• Mortality varied nearly fourfold among older LIC-born men by socioeconomic position.
• Hospitalisation gaps in older LMIC- and Swedish-born men largely vanished by wave 4.
• Temporal shifts in intersectional risk challenge static views of vulnerability.

COVID-19 caused devastating global mortality (World health organization, 2024), including substantial loss of lives in Sweden (Socialstyrelsen (The National Board of Health and Welfare), 2025). Yet, the burden of severe disease and mortality was unevenly distributed across populations. The early slogan ‘*we're all in this together’* obscured profound social inequalities in both morbidity and mortality (Bowleg, 2020). Although the acute phase of the pandemic has been declared over (Regeringskansliet, 2022; Williams et al., 2023), understanding its unequal impact remains essential, as pandemic preparedness that fails to integrate equity, risks reproducing and exacerbating the pre-existing structural inequalities that shaped pandemic outcomes.

Throughout the pandemic, there were persistent calls to identify and protect so-called ‘vulnerable groups’ (Sam, 2020). In Sweden, Tegnell et al. (2023) concluded that the national response flattened the epidemic curve but was less successful in safeguarding these groups (Tegnell et al., 2023). Sweden is a distinct setting in this regard. Compared with several other European countries, its early pandemic response relied more heavily on voluntary recommendations and individual responsibility. At the same time, its preparedness framework formally recognised the importance of ‘vulnerable groups’ and broader social determinants of health. This makes Sweden a valuable context for examining how vulnerability evolved over time across intersecting social positions, while also raising important questions about the dynamics of vulnerability: who is considered vulnerable, why, and in relation to what (Sam, 2020). Although SARS-CoV-2 is a virus, social conditions shaped both its spread and the unequal consequences of COVID-19 (Napier, 2020). Scholars have therefore described COVID-19 as a *syndemic*, emphasising how viral exposure interacts with pre-existing medical and social inequalities to influence the distribution, progression, and severity of disease (Bambra et al., 2020; Gravlee, 2020; Horton, 2020; Singer, 2020).

Inequalities in COVID-19 outcomes have been consistently observed across multiple social dimensions, including education, income, gender, occupation, disability status, racialized or ethnic minority, migrant status, and geographical area (Bambra et al., 2021; Chen et al., 2021; Fallah-Aliabadi et al., 2022; Kamalakannan et al., 2021; Khanijahani et al., 2021; McGowan & Bambra, 2022; Norström & Ramstedt, 2024; Saarinen et al., 2022). These patterns indicate that the pandemic largely followed existing social fault lines, with risks of severe disease and death unevenly distributed across social groups.

Conventional epidemiological studies have deepened our understanding of disease patterns, but the traditional risk-factor approach has been criticised for relying on *average effects* that obscure heterogeneity within a population (Merlo, 2014; Merlo et al., 2017). Focusing on mean risks limits the ability to identify who will experience an outcome and can misdirect interventions (Merlo et al., 2017). Narrow, single-factor analyses may fragment individuals into isolated categories, masking how multiple dimensions of social position intersect to shape lived experience and health outcomes (Bauer et al., 2021; Bowleg, 2012). Such models may also unintentionally reinforce essentialist or stigmatising narratives of ‘high-risk’ or ‘vulnerable’ groups (Chae et al., 2021; Merlo et al., 2017). For example, identifying racial or ethnic classifications as stand-alone risk factors risks diverting attention from the structural processes producing vulnerability (Massion et al., 2022).

A limited theoretical grounding in epidemiology continues to constrain efforts to capture the complexity of health inequalities (Bauer et al., 2021; Krieger, 2024). *Intersectionality* provides a framework for addressing some of these limitations by analysing social positions as co-constructed by multiple dimensions that are shaped in everyday life by interconnected systems of privilege and oppression at structural levels (Bowleg, 2012). Repeated calls have urged the application of intersectionality to increase nuance in understanding inequalities in COVID-19 (Bowleg, 2020; Maestripieri, 2021; Sasser et al., 2021). A growing body of research has responded to this call by examining COVID-19 through an intersectional perspective and highlighting how overlapping social positions are associated with heterogeneous health and social outcomes (Pathak et al., 2022; Riley et al., 2025; Rushovich et al., 2021; Salami et al., 2025). However, much of this research has been conducted in North America, and there is increasing recognition that variations in societal contextual and pandemic responses require context-specific analyses.

In Sweden, a recent MAIHDA study by Honkaniemi and Juárez (2026), identified intersectional inequalities in severe COVID-19 outcomes, challenging analysis of single identity dimensions in isolation. The present study complements this research by examining how such inequalities evolved over time across four pandemic waves in Sweden, defined according to periodisation commonly used by the Public Health Agency of Sweden and reflecting the dynamic nature of the pandemic. It further applies a more detailed operationalisation of intersectional strata, particularly regarding migration background and relative poverty. A more detailed understanding of the changing nature of health inequalities is crucial for strengthening the conditions for informing equitable, context-sensitive, and adaptive public health responses.

## 1 Intersectionality—Theoretical Foundations and epidemiological application

The concept of intersectionality was articulated within Black feminist thought as a critical response to analytical frameworks that reduced discrimination to singular dimensions or additive combinations, failing to account for their mutually constitutive nature (Crenshaw, 1989, pp. 138–167). Intersectionality emphasises the interdependent and dynamic nature of socially constructed identities and positions, examining how categories such as gender, class and migration background interact, and mutually shape one another, within systems of privilege and oppression, to produce both marginalisation and advantage (Hankivsky, 2012). Reducing individuals to single identity markers obscures this complexity as some intersections compound vulnerability while others confer protection.

Although intersectionality in public health has primarily been explored through qualitative research, interest in quantitative applications has grown (Bauer, 2014; Evans et al., 2018; Fisk et al., 2021; Hancock, 2007; Kapilashrami et al., 2015; Kapilashrami & Hankivsky, 2018). However, a systematic review found that many studies struggle to operationalise intersectionality's theoretical core (Bauer et al., 2021). Quantitative intersectional research has often relied on interaction terms in regression models or cross-stratified analyses, approaches that have generated important evidence on intersecting health inequalities in COVID-19 outcomes (Pathak et al., 2022; Rushovich et al., 2021; Salami et al., 2025). However, when many intersecting dimensions are analysed simultaneously, saturated interaction models may become unstable in sparse strata, and cross-stratified comparisons across large numbers of cells increase the risk of multiple-testing bias. *The Multilevel Analysis of Individual Heterogeneity and Discriminatory Accuracy (MAIHDA)* addresses some of these limitations by modelling variation both within and between intersectional strata, defined as combinations of social positions (Evans et al., 2018). Its random-effects structure partially pools estimates across strata, producing more stable predictions for small or sparsely populated combinations, while its *discriminatory accuracy* component assesses the extent to which intersectional strata distinguish individuals with higher risk, thereby linking population-level structure with individual-level heterogeneity (Bauer et al., 2021; Evans et al., 2018). MAIHDA can also decompose between-stratum variance into additive and residual intersectional components beyond the additive effects of included social positions. Applied within an intersectional framework, it moves beyond identifying isolated risk factors to examine how social hierarchies become embodied in health (Bauer et al., 2021; Evans et al., 2018, 2024). However, as Bowleg (2025) emphasises, intersectionality is not merely a tool for empirical investigation, but a framework that challenges simplifications and encourages critical reflection on how research questions, assumptions, and methods shape understandings of intersecting social positions and their relationship to interlocking systems of power, privilege, and oppression that produce health inequalities, thereby supporting more equitable public health practice (Bowleg, 2025).

## 2 Inequalities in COVID-19 – the relevance and meaning of ‘vulnerability’

The unequal distribution of COVID-19 outcomes has often been discussed through the lens of *vulnerability*, with terms such as ‘vulnerable groups’ or ‘vulnerable populations’ widely used (Altmejd et al., 2023; Khanijahani et al., 2021). Yet, the concept has been criticised for ambiguity and inconsistent use (Chae et al., 2021; Diderichsen et al., 2019). Vulnerability and susceptibility are often conflated (Chae et al., 2021) and labelling groups as vulnerable may reinforce reductionist explanations or obscure the structural processes producing inequality (Chae et al., 2021; Kapilashrami & Hankivsky, 2018). To clarify the concept, Diderichsen et al. (2019) distinguished between differential exposure, differential susceptibility, and differential capacity to buffer or avoid exposure, as well as to mitigate adverse outcomes (Diderichsen et al., 2019).

This dynamic nature of vulnerability was evident during COVID-19, as groups considered vulnerable shifted over time, while policies, viral variants, and social conditions evolved (Napier, 2020; Sam, 2020). Albani et al. (2022) likewise noted that susceptibility may also arise from chronic stressors linked to social disadvantage, even without underlying disease (Albani et al., 2022). Patterns of vulnerability were also shaped by the vaccine roll-out and immunity from prior infection. Understanding vulnerability as socially produced, context-dependent, and mutable is therefore crucial for interpreting health inequalities during and beyond the pandemic.

## 3 Previous research on inequalities in COVID-19 in Sweden

By March 2022, when Swedish authorities declared the acute phase of the pandemic over, nearly 100,000 individuals had been hospitalised and about 17,000 had died in a country with just above ten million inhabitants (Socialstyrelsen (The National Board of Health and Welfare), 2022). Of the four pandemic waves in Sweden, the first two waves peaked in April and December 2020, followed by a third wave linked to the Alpha variant in spring 2021 during the early vaccination rollout. A fourth wave emerged in late 2021, partly driven by the Omicron variant (Tegnell et al., 2023). Throughout the pandemic, Swedish authorities implemented varying public health measures aimed at reducing transmission, including physical distancing recommendations, limits on public gatherings, remote work recommendations, and expanded national testing, and later large-scale vaccination campaigns (Public Health Agency of Sweden (PHAS), 2023).

Advanced age was the primary risk during the pandemic, with almost 90% of fatalities occurring among those aged 70 years or older (Socialstyrelsen (The National Board of Health and Welfare), 2022). As elsewhere, Swedish studies identified marked disparities in severe COVID-19 outcomes. Elevated risks have been documented for men (Drefahl et al., 2020), individuals with lower income or education (Drefahl et al., 2020; Gustafsson et al., 2022; Norström & Ramstedt, 2024), migrants (Gustafsson et al., 2022; Rostila et al., 2023), older persons in multigenerational households (Brandén et al., 2020), residents of densely populated neighborhoods with high proportions of immigrants (Fonseca-Rodríguez et al., 2021), and workers in occupations involving frequent interpersonal contact (Alderling et al., 2021). A recent Swedish study moved beyond single-dimensional approaches and identified intersectional inequalities in severe COVID-19 outcomes across the pandemic period as a whole. The highest risks were observed among older, low-income, men from the Global south and the lowest among younger, high-income Swedish-born women (Honkaniemi & Juárez, 2026).

The present study complements this research by examining how such inequalities shifted across the four pandemic waves in Sweden, thereby capturing the dynamic nature of vulnerability across changing epidemiological and societal contexts. It further adopts a more granular operationalisation of social strata, particularly regarding migration background, while also including measures of relative poverty, education, gender and age.

### 3.1 Aim

This study applied an intersectional framework to analyse how the COVID-19 pandemic unfolded over four waves in Sweden, focusing on how overlapping social positions—age, gender, income, educational attainment, and migration background—shaped vulnerability and resilience in relation to severe outcomes, including hospitalisation and mortality.

The study was guided by the following research questions.

1. How does an intersectional MAIHDA approach, compared with single-axis analyses, alter the identification and interpretation of inequalities in severe COVID-19 outcomes?
2. How did patterns of inequality across intersectional strata in hospitalisation and mortality vary, by pandemic wave and age group?

### 3.2 Methods

To address this aim, we conducted a nationwide, register-based cohort study covering the entire adult population of Sweden, linking individual-level sociodemographic data with COVID-19 outcomes across four pandemic waves (2020-2022). This design enabled us to examine how multiple axes of social position jointly shaped patterns of COVID-19 hospitalisation and mortality. The nationwide design provided coverage and statistical power, while *longitudinal follow-up* across all four waves allowed us to assess how inequalities evolved over time.

#### 3.2.1 Study population

The study utilised a dynamic cohort comprising the entire Swedish population aged 18 years or older, followed from March 2020 to March 2022. This period corresponds to the four pandemic waves identified by the Swedish National Board of Health and Welfare and the Public Health Agency of Sweden: wave 1 (1 March 2020—30 September 2020), wave 2 (1 October 2020—31 January 2021), wave 3 (1 February 2021—30 June 2021), and wave 4 (1 July 2021—31 March 2022) (Tegnell et al., 2023). We used *SCIFI-PEARL* (*Swedish COVID-19 Investigation for Future Insights—a Population Epidemiology Approach using Register Linkage*), a nationwide multi-register platform designed for investigating COVID-19 in Sweden (Nyberg et al., 2021). The SCIFI-PEARL database integrates multiple national and regional registers, such as: *SmiNet* (the national database of notifiable diseases), *National Patient Register* (hospitalisation and diagnosis data), *Cause-of-Death Register* (providing mortality data), and *Longitudinal Integrated Database for Health Insurance and Labour Market Studies (LISA)* (demographic and socioeconomic information) (Ludvigsson et al., 2019). For each pandemic wave, the population at risk for COVID-19 outcomes included individuals aged 18 years or older, adjusted for the dates they turned 18, immigrated to or out-migrated from Sweden, or died.

#### 3.2.2 Outcome variables

The study outcomes were COVID-19 hospitalisation and mortality, identified by a primary diagnosis of COVID-19 using the *International Classification of Diseases,* 10th *Revision (ICD-10)* codes U07.1 or U07.2.

#### 3.2.3 Exposure variables

Exposure variables included age, sex, income, education level, and birthplace income classification (defined below), based on previous evidence identifying them as predictors of severe COVID-19 outcomes (see below). While age reflects both biological susceptibility and socially patterned life circumstances, the other variables primarily capture socially structured forms of vulnerability, including inequalities in exposure, susceptibility through comorbidity or adverse living conditions, and access to protective resources (Diderichsen et al., 2019).

Analyses were stratified by adults aged 18-69 and ≥ 70, as age was a major determinant of severe outcomes. *Sex* was defined from register data (women and men). Income was dichotomised using the *at-risk-of-poverty* threshold—equivalised individual disposable annual income below or above 60% of the national median (Eurostat, 2025), with data from the *LISA register*. The corresponding 60% cut-off values were 203,280 SEK in 2019, 206,340 SEK in 2020 (Waves 1–2), 239,040 SEK in 2021 (Waves 3–4), and 246,240 SEK in 2022. Education, also from *LISA*, captures dimensions of social advantage and power and is associated with COVID-19 mortality in Sweden (Norström & Ramstedt, 2024). *Educational attainment* was dichotomised into low (primary and upper secondary education) and high (university, college, or post-graduate education). Country of birth has been associated with severe outcomes, with higher risks consistently reported among non-Swedish-born groups (Aradhya et al., 2021; Juárez et al., 2024; Rostila et al., 2023). These risks stem from differential exposure (Aradhya et al., 2021), higher prevalence of underlying conditions (Juárez et al., 2024; Rostila et al., 2023), and socioeconomic disadvantages (Tinghög et al., 2007) including relative poverty (Fritzell et al., 2011). Migrant status may also entail increased susceptibility resulting from trauma or discrimination, compounded by adverse conditions in the host country, including limited access to protective resources. However, migrant status encompasses substantial heterogeneity (Fisk et al., 2021; Yaya et al., 2020), making broad categorisations analytically limiting. Although not perfect, this study addressed this by defining *‘Birthplace Income Classification’* utilising the *World Bank classification of country income levels* (World Bank, 2024), distinguished individuals born in Sweden, high-income countries (HIC), upper middle-income countries (UMIC), lower middle-income countries (LIMC), and low-income countries (LIC). Analyses were stratified by age group (18-69 and >=70 years), with intersectional strata defined by combinations of sex, income, education, and ‘Birthplace Income Classification’, resulting in 40 strata per age group.

#### 3.2.4 Statistical analyses

This study examined intersectionality inequalities using the *Multilevel Analysis of Individual Heterogeneity and Discriminatory Accuracy (MAIHDA)* approach (Evans et al., 2018, 2024; Merlo, 2014; Wemrell et al., 2017), a robust method in social epidemiology for analysing health disparities (Merlo, 2018). Our analytical strategy followed Fiske et al. (2018) and a recent *I-MAIHDA* tutorial (Evans et al., 2024). We conducted separate analyses for COVID-19 hospitalisation and mortality across four pandemic waves, stratifying age (18-69 and ≥ 70 years). Using two sequential multilevel logistic regression models, we estimated predicted incidence rates and 95% confidence intervals (CIs).

*Model 1* (*Simple intersectional model)—*This unadjusted random-intercepts model nested individuals within intersectional strata to estimate variance components. We calculated the *Intraclass Correlation Coefficient (ICC)* to quantify discriminatory accuracy—i.e., the proportion of total variance attributable to intersectional strata. Model 1 also provided stratum-specific predicted incidence rates and 95% CIs using marginal standardization, enabling comparison of intersectional inequalities across waves.

*Model 2* (*Intersectional interaction model*)– *Model 2* included all four dimensions defining intersectional strata within each age group as fixed main effects. If no interactions existed between dimensions, *Model 2* would fully explain all between-stratum variance, reducing random effects to zero. Any remaining variance therefore reflects intersectional interactions. This model also produced mutually adjusted unidimensional odds ratios (ORs) for sex, income, education, and birthplace income classification. Predicted incidence rates (per 10,000 population) were generated from model-based marginal predictions incorporating both fixed and random components.

## 4 Results

During the study period, Sweden recorded 67,997 COVID-19 hospitalisations and 15,895 deaths among individuals aged 18 and older (Table 1), corresponding to 84.3 hospitalisations and 19.7 deaths per 10,000 population. Supplementary Table 1 presents absolute counts, and Supplementary Table 2 reports incidence per 10,000, across the four pandemic waves, stratified by age and sociodemographic characteristics.

**Table 1:** Number of individuals with COVID-19 hospitalisations and mortality during the four pandemic waves in Sweden, stratified by age group and sociodemographic and economic characteristics.

Variables	18 - 69 years old					≥70 years old				
	Total population at risk	COVID-19 hospitalisations		COVID-19 deaths		Total population at risk	COVID-19 hospitalisations		COVID-19 deaths	
		Number of cases	Rates per 10,000 population	Number of cases	Rates per 10,000 population		Number of cases	Rates per 10,000 population	Number of cases	Rates per 10,000 population
**Total**	6,474,223	36,636	56.6	1748	2.7	1,590,784	31,361	197.1	14,147	88.9

**Sex**										
Women	3,184,792	14,323	45.0	494	1.6	861,320	13,971	162.2	6516	75.7
Men	3,289,431	22,313	67.8	1254	3.8	729,464	17,390	238.4	7631	104.6

**Birthplace income classification**										
Swedish	4,974,546	20,190	40.6	1096	2.2	1,403,388	24,996	178.1	11,797	84.1
HIC^a^	476,527	4031	84.6	199	4.2	148,200	4187	282.5	1757	118.6
UMIC^a^	463,767	6197	133.6	203	4.4	22,637	1276	563.7	340	150.2
LMIC^a^	245,709	2728	111.0	94	3.8	11,659	507	434.9	142	121.8
LIC^a^	313,674	3490	111.3	156	5.0	4900	395	806.1	111	226.5

**Education**										
High Education	2,715,025	11,873	43.7	398	1.5	424,927	6351	149.5	2391	56.3
Low Education	3,759,199	24,763	65.9	1350	3.6	1,165,857	25,010	214.5	11,756	100.8

**Income**										
High Income	4,232,772	21,829	51.6	798	1.9	470,657	8037	170.8	3339	70.9
Low Income	2,241,452	14,807	66.1	950	4.2	1,120,127	23,324	208.2	10,808	96.5

a High-income countries (HIC), Upper-middle-income countries (UMIC), Lower-middle-income countries (LMIC), Low-income countries (LIC).

Table 1 shows that hospitalisation incidence was, as expected, higher among individuals aged ≥70 than among those aged 18–69 (197 vs. 57 per 10,000, respectively). A similar pattern was observed for mortality, with rates of 88.9 per 10,000 among older individuals compared with 2.7 per 10,000 among younger adults. Mortality rates were consistently higher among men, migrants from low-income countries (relative to those from high-income countries or Swedish-born), and individuals with low income or low education in both age groups.

During wave 1, hospitalisation incidence among migrants aged ≥70 years from LICs was roughly four times higher (282.5 per 10,000) than among counterparts from HICs (63.9 per 10,000) (Supplementary Table 2). Differences across birthplace income classifications varied by wave and were generally less pronounced among younger adults. Low income was associated with increased hospitalisation and mortality for waves 1, 2, and 4 for both age groups. In wave 3, differences by income were small; among older adults, mortality patterns slightly reversed, with the high-income group showing marginally higher rates.

### 4.1 Intersectional strata characteristics and COVID-19 hospitalisation and mortality

Fig. 1 displays the odds ratios (ORs) and 95% confidence intervals (CIs) for COVID-19 hospitalisations and mortality by sociodemographic variables defining the intersectional strata, across the four pandemic waves and stratified by age groups (18-69 and ≥ 70) and by sociodemographic variables defining the intersectional strata. Although the direction of associations was generally consistent across age groups, the magnitude of ORs, the width of CIs, and statistical significance varied, particularly between hospitalisation and mortality outcomes.

**Fig. 1:**
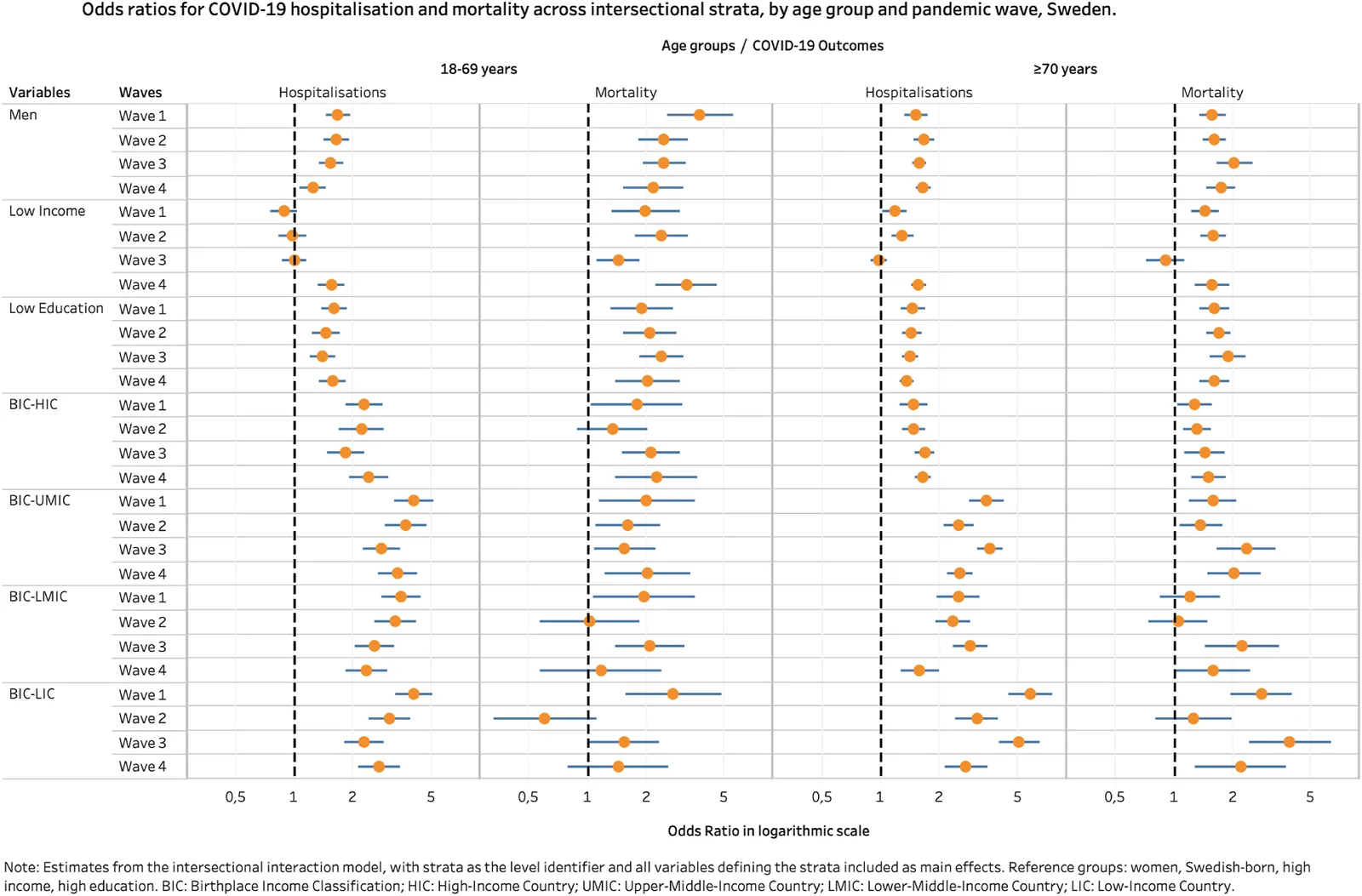
Odds ratios for COVID-19 hospitalisation and mortality across intersectional strata, by age group and pandemic wave, Sweden. Note: Estimates from the intersectional interaction model, with strata as the level identifier and all variables defining the strata included as main effects. Reference groups: women, Swedish-born, high income, high education. BIC: Birthplace Income Classification; HIC: High-Income Country; UMIC: Upper-Middle-Income Country; LMIC: Lower-Middle-Income Country; LIC: Low-Income Country.

Male sex, low education, and birthplace income classification (across all migrant categories) were associated with a higher likelihood of hospitalisation relative to their respective reference groups in both age groups. While all migrant categories showed elevated risks compared with Swedish-born individuals, substantial variation existed between the four birthplace income classification categories.

Patterns of inequality in COVID-19 mortality broadly mirrored those observed for hospitalisation, though CIs were typically wider, resulting in fewer statistically significant associations (Fig. 1). Male sex and low education were consistently linked to increased mortality across all waves and in both age groups. The association between low income and mortality differed by age group and wave: among younger adults (18-69), low income showed no association with mortality during waves 1-3 but was positively associated in wave 4. Among older age group (≥70), low income was associated with higher mortality during waves 1, 2 and 4, but not in wave 3. For younger individuals, all migration categories generally had higher mortality risk, although associations were not consistently statistically significant. For example, no differences were observed for migrants from HIC in wave 2 or from LMICs and LICs in waves 2 and 4. A similar pattern was observed among the older adults, with no statistically significant mortality differences between Swedish-born individuals and migrants from LMICs during waves 1 and 2, or from LICs during wave 2 (Fig. 1).

### 4.2 Intraclass correlation in multilevel analysis of COVID-19 hospitalisations and mortality

The *Intraclass Correlation Coefficient (ICC)* quantifies the proportion of total variance attributable to intersectional strata, and the results were stratified by age group and outcome. In the null models, ICC values varied by age and outcome. For mortality among adult under 70 years, ICC values were 25% in wave 1, 15% in wave 2, 17% in wave 3, and 22% in wave 4. These findings suggest that the intersectional strata explained a considerable proportion of mortality variance among younger individuals. Hospitalisation outcomes showed a similar ICC pattern for both age groups: values ranged between 7% and 13% among the younger group and 9% to 14% among the older group (Supplementary Table 3).

### 4.3 Predicted incidence of COVID-19 hospitalisations and mortality across intersectional strata

Fig. 2 shows predicted incidence rates per 10,000 population for COVID-19 hospitalisations and mortality across 80 intersectional strata, comprising 40 strata for adults aged 18-69 years and 40 for those aged ≥70 years, across the four pandemic waves. Each row represents one stratum defined by sex, birthplace income classification, income, and education. Cells are colour-coded by rank within each wave and age group, from the lowest incidence (green) to the highest (red), allowing the reader to identify which combinations of social positions concentrated the highest and lowest risks at each point in the pandemic. The figure can be read in three ways. Reading across a row shows how a given stratum's relative position shifted across waves. Reading down a column within a wave shows which strata clustered at the high and low ends of the distribution at that point. Comparing the left (18–69) and right (≥70) panels shows how the patterning of inequality differed by age group.

**Fig. 2:**
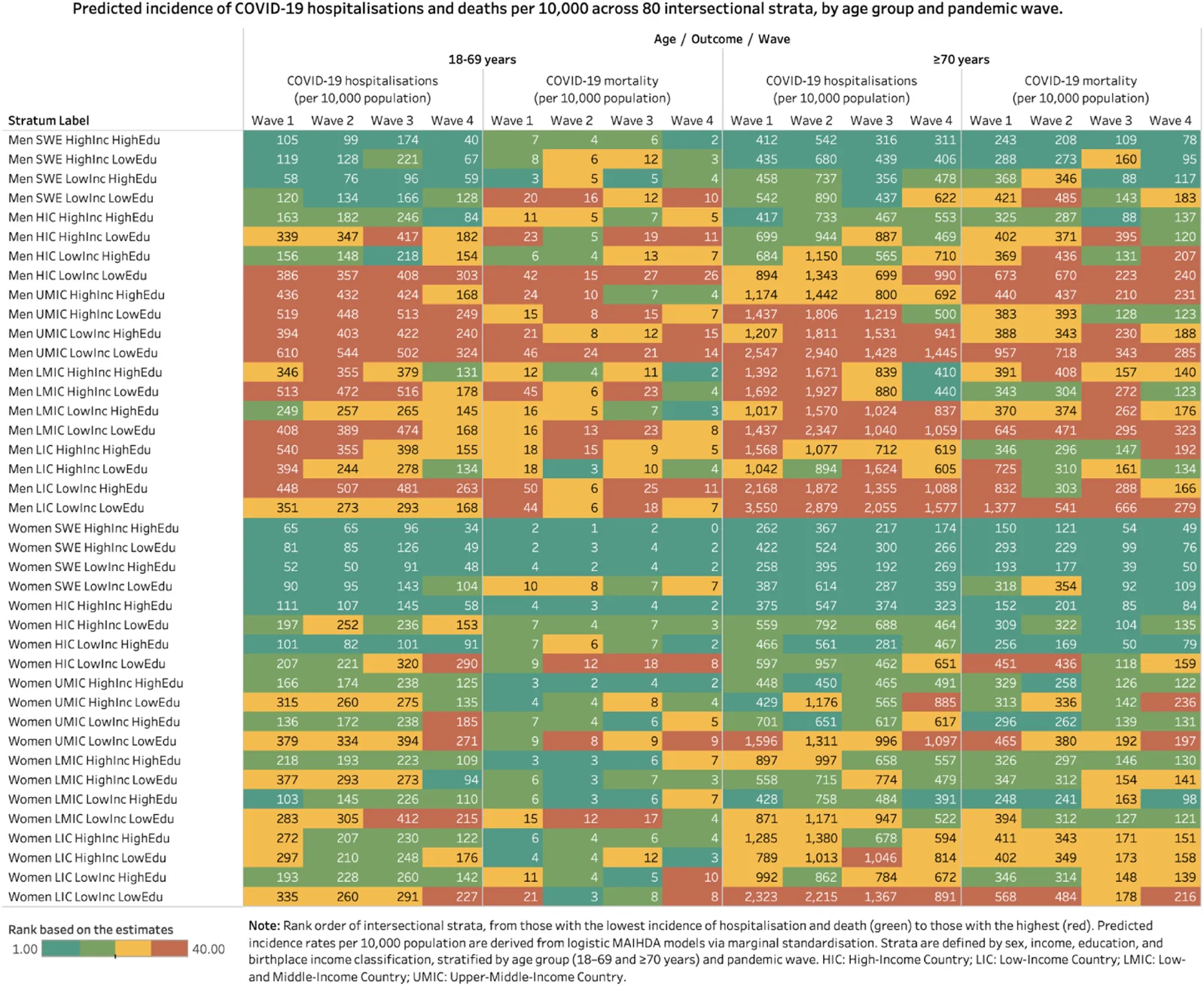
Predicted incidence of COVID-19 hospitalisations and deaths per 10,000 across 80 intersectional strata, by age group and pandemic wave. Note: Rank order of intersectional strata, from those with the lowest incidence of hospitalisation and death (green) to those with the highest (red). Predicted incidence rates per 10,000 population are derived from logistic MAIHDA models via marginal standardisation. Strata are defined by sex, income, education, and birthplace income classification, stratified by age group (18-69 and >=70 years) and pandemic wave. HIC: High-Income Country; LIC: Low-Income Country; LMIC: Lower-_Middle-Income Country; UMIC: Upper-Middle-Income Country.

As expected, incidence rates were substantially higher among older adults for both outcomes. Within each age group, however, intersectional inequalities varied substantially by wave and outcome, and no single stratum consistently occupied the highest-risk position across all waves. The detailed patterns by age group and outcome are described below.

### 4.4 Younger group (18-69 years) — hospitalisations

Strata with Swedish-born individuals and women generally showed lower hospitalisation incidence, though notable variation existed. In wave 1, incidence among women ranged from 52 per 10,000 (Swedish-born, low income, high education) to 379 per 10,000 (UMIC-born, low income, low education). Among migrant men, rates ranged from 156 per 10,000 (HIC-born, low income, high education) to 610 per 10,000 (UMIC-born, low income, low education).

During the first two waves, the ten highest-incidence strata comprised exclusively migrant men. However, by wave 3, UMIC-born women (low income, low education) also appeared among the highest-incidence strata, and by wave 4, migrant women occupied five of the ten highest-incidence strata. Despite migrant men consistently having high hospitalisation rates, substantial within-group variation was evident. For example, by wave 4, incidence of hospitalisation among migrant men ranged from 84 per 10,000 (HIC-born, high income, high education) to 324 per 10,000 (UMIC-born, low income, low education).

### 4.5 Younger group (18-69 years) – mortality

Similar to hospitalisations, the COVID-19 mortality rates were typically lower among Swedish-born individuals and women. However, intersectional comparisons showed greater diversity across strata and waves compared to hospitalisations. Most strata with the highest mortality comprised migrant men, although Swedish-born men with low income and education appeared among the top ten in three of the four waves. Several strata with women also featured prominently in the highest mortality groups. Mortality incidence exhibited greater fluctuation across waves than hospitalisation. For example, among LIC-born men with low income and education, mortality declined markedly from 44 per 10,000 in wave 1 to 6, 18, and 7 per 10,000 in subsequent waves. In wave 2, their incidence rate was notably lower than rates observed in some migrant women and Swedish-born men with low income and education. A similar decline occurred among LIC-born women with low income and education, with mortality falling from 21 per 10,000 in wave 1 to 3 per 10,000 in wave 2.

### 4.6 Older group (≥70) – hospitalisation

In the older group, Swedish-born individuals and women also exhibited lower hospitalisation rates. LIC-born men with low income and education consistently ranked among the highest-incidence strata, with rates as high as 3550 per 10,000 in wave 1, declining to 2,879, 2,055, and 1577 per 10,000 in subsequent waves. Nevertheless, incidence and rankings varied across strata and waves. In wave 4, LMIC-born men with high income and low education had hospitalisation rates comparable to their Swedish-born counterparts (440 vs 406 per 10,000, respectively).

LIC-born women with low income and education consistently ranked within the ten highest hospitalisation strata, notably appearing among the top four during waves 1 and 2. Substantial variations existed within migration categories. For instance, in wave 1, hospitalisation incidence among LIC-born men was over three times higher in those with low income and low education (3550 per 10,000) compared to those with high income and low education (1042 per 10,000), a pattern that persisted across subsequent waves.

#### 4.6.1 Older group (≥70)–mortality

Intersectional inequalities in mortality were more variable across strata and waves compared with hospitalisation. Generally, mortality rates were higher among migrants and men, but this pattern had notable exceptions. For instance, LIC-born men with high income and high education had relatively low mortality (296 per 10,000 in wave 2), lower than several Swedish-born and HIC-born male strata. Within migrant groups, variation was also substantial: among LIC-born men, mortality ranged from 346 per 10,000 to 1377 per 10,000 in wave 1, though this range narrowed considerably by wave 4 (134 per 10,000 to 279 per 10,000).

### 4.7 Interaction effects of the predicted proportion of COVID-19 hospitalisations and mortality

Supplementary Fig. 1 presents the interaction effects for intersectional strata on predicted incidence of COVID-19 hospitalisation and mortality across the four pandemic waves, stratified by age (18-69 and ≥ 70). Only three significant interactions were identified among older individuals, whereas statistically significant interactions were more common among younger individuals, especially concerning hospitalisation outcomes, with the most pronounced interaction effects observed among younger LIC-born men. These results indicate that most inequalities in COVID-19 hospitalisation and mortality reflected additive effects of social position, with few statistically significant interactions.

### 4.8 Single-axis versus intersectional identification of inequality

Single-axis analyses indicated higher risks of COVID-19 hospitalisation and mortality among men, individuals with low income or education, and migrants. In contrast, MAIHDA analyses revealed substantial heterogeneity within these categories and wave-specific re-ordering of risk across intersectional strata. Several strata conventionally classified as disadvantaged exhibited risks comparable to more advantaged strata, while a limited number of specific intersections concentrated the highest incidence. ICC estimates further showed that intersectional strata explained a meaningful share of outcome variation, particularly for mortality among adults under 70 years.

## 5 Discussion

### 5.1 Key contributions and framing

Our findings demonstrate that severe COVID-19 outcomes cannot be adequately understood without considering how multiple dimensions of social position—such as age, gender, socioeconomic status, and migration background—intersect and evolve over time. By analysing all four pandemic waves, each marked by distinct epidemiological conditions and policy contexts, our study highlights how intersectional inequalities shifted across time and context, underscoring the need for adaptive, equity-focused public health responses.

This study makes three key contributions. First, it empirically demonstrates that intersectional inequalities in hospitalisation and mortality changed across pandemic waves, underscoring the temporal fluidity of vulnerability. Second, it complements previous analyses by applying more granular intersectional strata. Third, it grounds the quantitative analysis in intersectionality theory, aiming to present results that can support critical dialogue and equity-oriented change, both of which are central to the framework (Bowleg, 2025; Hankivsky, 2012).

### 5.2 The relevance of intersectional strata and the central role of age

Intersectional strata explained a considerable share of the variance in COVID-19 outcomes, particularly among younger individuals and for mortality, with *ICCs* reaching from 15 to 25%. Among older individuals, *ICCs* were typically below or just above 10%, which may partly suggest the greater importance of biological susceptibility and frailty at older ages. This was somewhat lower than the VPCs reported in the time-combined analyses by Honkaniemi and Juárez (2026). This difference may partly reflect methodological differences, including our wave-specific analyses and age-stratified models, which capture greater temporal heterogeneity and reduce the contribution of broad age-related differences to between-stratum variance.

The patterns observed in the present study indicate how the relative contribution of social versus biological mechanisms may shift with age, aligning with previous research on age-related immune susceptibility (Bajaj et al., 2021). As most COVID-19 deaths in Sweden occurred among individuals aged 70 years or older (Socialstyrelsen (The National Board of Health and Welfare), 2025), biological susceptibility and frailty may have become more dominant in this group, thereby reducing the relative contribution of other, primarily socially structured, positions to outcome variation. In younger age groups, by contrast, inequalities across intersecting social positions may more clearly reflect socially structured differences in exposure, susceptibility through comorbidity or adverse living conditions, and access to protective resources. Consequently, studies that rely solely on age adjustment (Juárez et al., 2024; Rostila et al., 2021, 2023) may underestimate the influence of social determinants, especially in younger populations. The prominence of age as a risk factor may be virus-specific, but it underscores the importance of understanding how pathogen-host interactions and social structures jointly shape inequalities. This insight has implications for pandemic preparedness, as future pathogens may disproportionately affect groups defined not only by age but also by their position within intersecting systems of disadvantage and privilege.

### 5.3 Additivity, interaction, and complexity of social positioning

The analyses primarily revealed additive effects, with few statistically significant interactions, particularly among older individuals. Interaction effects were more common among younger individuals, particularly for hospitalisation. Drawing on Evans et al. (2024), informed by feminist theory and King's typology, our findings indicate to a predominant additive pattern, (‘*double jeopardy*’), where each marginalised position adds to disadvantage. In contrast, *‘multiple jeopardy’* denotes compounded effects beyond additivity (Evans et al., 2024).

One notable interaction emerged among younger men born in LICs with high educational attainment but low income, where the protective effect of education reversed in intersectional models. This pattern may reflect the well-documented occupation–education mismatch experienced by many migrants in Sweden (Irastorza & Bevelander, 2021), whereby highly educated immigrants are over-represented in essential, high-risk occupations, increasing exposure to SARS-CoV-2 and the risk of severe outcomes (Rosengren et al., 2022). From a public health perspective, even additive findings remain informative for identifying groups facing disproportionate disadvantage and vulnerability (Fisk et al., 2018). Previous studies report elevated ICU admission and mortality risks among migrants from broadly labelled *‘African’* or *‘Middle Eastern’* regions (Rostila et al., 2021, 2023). Our findings refine this evidence, showing that such regional categories obscure substantial within-group heterogeneity, reinforcing the need for more nuanced, intersectionality-informed analyses.

### 5.4 Comparative evidence and need for theoretical clarity

The complexity of our findings challenges conventional binary conceptions of social positioning–for example, the simple contrast between *‘migrant’* and *‘native’*—and is consistent with previous research demonstrating substantial heterogeneity within such categories (Fisk et al., 2021; Krasnik, 2015; Mulinari et al., 2015). Mortality among older men born in low-income countries varied nearly fourfold during the first wave (346 vs. 1377 per 10,000), and in some migrant strata mortality was lower than among certain Swedish-born strata. Swedish-born men with low income and education also consistently appeared among the highest-risk strata in three of the four waves. Detecting such heterogeneity across 80 strata, including small cells defined by migrant background, income, and education in combination, was made possible by MAIHDA's partial pooling, which stabilises estimates that would be unreliable in saturated interaction models or single cross-stratified tables.

Although recent Swedish studies employed more refined categorisations of country of birth—by global region (Honkaniemi & Juárez, 2026; Juárez et al., 2024; Rostila et al., 2021, 2023), or the World Bank's country income level classification (Gustafsson et al., 2022), most analyses still primarily adjust for age, sex, and socioeconomic status (Juárez et al., 2024; Kjøllesdal et al., 2023; Rostila et al., 2021, 2023). Such practices risk masking differences in structural conditions (Merlo et al., 2017). In particular, broad regional categories of birthplace can obscure meaningful social and historical variation, as well as considerable heterogeneity within these groups (Rostila et al., 2023). As scholars in cultural geography note, the regions often used to classify birthplace are historical and political constructs rather than natural entities. Lewis & Wigen's *The Myth of Continents* (Lewis & Wigen, 1997), for example, demonstrates how regional labels such as *‘Africa’* or *‘Asia’* reflect historically and politically situated classifications rather than natural entities. This critique has received limited attention in epidemiology, where categories linked to birthplace are often highly aggregated. A recent systematic review of COVID-19 mortality among migrants and ethnic-minority populations highlighted this gap, noting that many studies used categories so broad that group-specific social conditions and histories relevant to health could not be considered (Mazzalai et al., 2023). Bauer et al. (2021) therefore emphasise the need for theoretical transparency in quantitative research, advocating that categorisations make explicit the power relations and assumptions underpinning them (Bauer et al., 2021). Similarly, Krieger (Krieger, 2024) has recently observed that epidemiology remains largely atheoretical.

A key consideration is that categorisations should be grounded in clear, context-specific reasoning about underlying mechanisms, because the explanations they generate have policy and practical implications. In this respect, our use of the World Bank's income classification of countries is far from optimal, as even a five-category classification inevitably obscures substantial heterogeneity and unequal living conditions within countries of origin. The substantial within-stratum variation observed in our analyses also indicates that our categorisation does not fully capture the sources of heterogeneity relevant to COVID-19 vulnerability: MAIHDA quantifies how much heterogeneity remains within categorical groupings, but cannot explain what produces these inequalities. Based on a recent review, increased exposure and limited access to resources among migrant groups may arise through conditions such as crowded housing, high-risk occupations, inadequate health information, and distrust in institutions (Hintermeier et al., 2024). These differences in exposure, living conditions, and access to health and social services may partly reflect structural discrimination and unequal power relations in both countries of origin and countries of settlement, embedded in labour markets, housing conditions, and institutional structures. Some groups may also face increased susceptibility due to adverse pre- and peri-migration conditions, although Hintermeier et al. (2024) conclude that important knowledge gaps remain regarding the role of such factors for COVID-19-related vulnerability.

### 5.5 Intersectionality and temporal fluidity of inequality

While recent Swedish MAIHDA analyses demonstrated substantial intersectional inequalities across the pandemic as a whole (Honkaniemi & Juárez, 2026), our wave-specific analyses suggest that risk patterns shifted considerably across pandemic phases. Our findings align with previous Swedish research showing that pandemic-related inequalities evolved over time according to migrant status (Rostila et al., 2023) and income (Gauffin et al., 2023). However, these temporal trends differed by social dimension. For instance, Rostila et al. (2023) found declining disparities in intensive-care admission between migrants and Swedish-born, with the largest differences during the first wave (Rostila et al., 2023), whereas Gauffin et al. (2023) reported widening income-based inequalities in intensive care over successive waves (Gauffin et al., 2023). Our wave-specific intersectional analyses extend this work by showing how inequalities shifted across pandemic phases. It reveals that changes in inequality over time were not uniform across strata: disparities either widened or narrowed across pandemic waves depending on the specific intersections. For instance, hospitalisation disparities between Swedish-born older men with high income and those born in upper-middle-income countries with high income, but low education decreased sharply in wave 4 compared to waves 1-3. These findings illustrate that vulnerability was temporally contingent and probably shaped by shifting epidemiological, policy, and social conditions, including changes in vaccination roll-out, and the emergence of new virus variants. From a policy perspective, this temporal fluidity underscores the need for pandemic preparedness and response strategies that can identify and adapt to changing patterns of risk and inequality. In line with Bowleg (2025), this study thus highlights that inequalities are not inherent in identity combinations themselves, but arise through the meanings and consequences these combinations acquire within specific temporal, political, and social contexts.

### 5.6 Structural vulnerability and Bio-Socio-temporal patterns

The inequalities observed across intersectional strata and pandemic waves reflect evolving viral exposure, differential immunity, and unequal access to protective resources —factors shaped by structural conditions and policy decisions. These patterns underscore that the pandemic's unequal impact was not inevitable but socially influenced (Napier, 2020). Despite substantial variation across strata and waves, some consistent patterns of disadvantage emerged, notably older men from low- and upper-middle-income countries and older women from low-income countries with low income and education. Among younger age groups, patterns varied more, particularly for mortality, which may reflect the disproportionate mortality among frail individuals in earlier waves, evolving virus dynamics associated with Alpha and Omicron, and unequal access or uptake of vaccination (Gauffin et al., 2023; Spetz et al., 2022).

Sweden's early response (The Public Health Agency of Sweden, 2019) relied heavily on voluntary recommendations, individual responsibility, and vaccination-based protection, which may have interacted differently with social and occupational conditions across groups. For example, some groups may have had fewer opportunities to work from home, limiting their ability to comply with recommendations. Earlier and more systematic integration of intersectional perspectives on vulnerability could therefore have strengthened pandemic planning by anticipating variation in exposure and vulnerability across social positions, supporting continuous monitoring of how these patterns develop over time.

As Diderichsen and da Silva's (Diderichsen & da Silva Augusto, 2021) argue in relation to Zika virus, the causes that generate inequalities in disease risk—such as differential exposure to infection—are not necessarily the same as those that generate inequality in the consequences of disease, including severity, access to care or social marginalisation. They also emphasise that *“The causes that need to be addressed to reduce inequalities of a disease might however be different from those needed to reduce its average occurrence”.* Addressing inequalities therefore requires advance pandemic planning and guidance that help policy- and decision-makers rapidly formulate measures sensitive to variations in living conditions, social circumstances, and pathogen characteristics. In infectious-disease pandemics, responses that fail to reach disadvantaged groups may sustain transmission, facilitate viral mutation, and ultimately increase population-level risk (Md Khairi et al., 2022).

Honkaniemi and Juárez (2026) recently concluded that observed inequalities supported the use of universal preventive measures, such as lockdowns and mask mandates, to protect “vulnerable populations”. While population-wide interventions can reduce inequalities by lowering overall transmission, our findings also highlight the importance of proportionate and adaptive interventions that recognise how vulnerability may have different starting points and change across intersecting social positions as pandemic phases evolve. However, intersectional analyses can identify inequitable patterns of risk; they cannot, by themselves, determine which specific public health interventions will be most effective for reducing them. Integrating intersectional perspectives into preparedness guidance may therefore support more responsive surveillance and response planning, helping decision-makers detect emerging unequitable patterns earlier and adapt recommendations and interventions as conditions change.

Given the dynamic and context-dependent nature of vulnerabilities, our findings do not predict specific future risk groups. Instead, they illustrate how vulnerability is fluid and shaped by interactions between the pathogen characteristics, host biology, social structures, and political context. This underscores the need for pandemic preparedness that anticipates changing risks and inequalities across time and social position. In practice, disease surveillance and public health interventions should be responsive to shifting inequalities across intersecting social positions and pandemic phases, rather than relying on static assumptions about “high-risk” groups. Ultimately, however, reducing the inequitable syndemic consequences of future pandemics requires not only intersectionality-informed surveillance and response, but also political commitment to addressing the structural conditions that produce unequal vulnerability.

## 6 Strengths, limitations and directions for future research

A key strength of this study is the use of nationwide, linked register data covering the entire adult population of Sweden, enabling analysis across four pandemic waves with minimal selection bias. The large sample size and complete follow-up enhanced statistical power to detect intersectional inequalities, while the MAIHDA framework provided a theoretically grounded approach to quantifying inequalities arising from both additive and emergent intersectional effects.

Several limitations warrant consideration. First, data on specific country of origin, migration reasons, or duration of residence were unavailable or too limited for inclusion, limiting insight into exposure and vulnerability contexts. Thus, although this study adopts a fairly granular operationalisation of migrant background, it still relies on broad categories that obscure the social and structural processes (including living conditions in country of origin, migration history, duration of residence, legal status, occupational conditions, experiences of discrimination and access to health and social care) that shape differential exposure and heightened susceptibility. The within-stratum heterogeneity we observe indicates that factors producing inequality remain unmeasured. Second, we did not have access to complete information on disability status or other physical and psychological characteristics. This is problematic given that disability may involve both biological susceptibility and socially structured. Third, we did not estimate the proportional contribution of specific strata to the total disease burden. While such estimates may support policy and resource allocation, they also require careful consideration of trade-offs between reducing incidence, promoting equity, and upholding the right to health.

Our models did not directly account for pandemic policies, such as vaccination or testing, which may vary across strata (Spetz et al., 2022) and influence vulnerability patterns. These omissions may reflect underlying structural inequalities but also limit interpretation of observed patterns.

The intersectional strata are analytical constructs rather than inherent risk groups or risk factors. Alternative migrant typologies—such as ethnicity or legal status—may have yielded different results. As Krieger (2024) argues, reducing social determinants to individual-level risks obscures systemic causes. Our aim was therefore not to identify categories of “vulnerable groups” but to illustrate how intersecting forms of disadvantage co-produce health outcomes. This underscores the need for careful interpretation and avoidance of overgeneralisation; adverse outcomes should not be attributed to group membership per se, but broader systems of discrimination and inequality (Evans et al., 2024).

All analytical approaches involve trade-offs in relation to intersectional theory. Our approach aligns with inter- and intra-categorical intersectionality approaches, but less so with anti-categorical approaches (McCall, 2005). While *MAIHDA* advances intersectional methods (Bauer et al., 2021), it relies on pre-defined categories. We acknowledge this limitation while building on recent efforts to embed intersectionality more fully into quantitative research design and interpretation (Evans et al., 2024).

Future studies should further investigate the structural conditions generating differential vulnerability, including inequalities in exposure, susceptibility, and capacity to respond (Diderichsen et al., 2019). Such work is essential for shifting public health away from crisis-driven responses toward addressing the structural root causes of syndemic dynamics—including disparities in living conditions, access to social protection, and pre-existing health burdens.

## 7 Conclusion

This study underscores the need for pandemic preparedness and response strategies that are sensitive to the intersectional and dynamic dimensions of health inequality. Applying an *intersectionality* perspective across the COVID-19 pandemic in Sweden, we reveal pronounced variations in hospitalisation and mortality across intersectional strata and over time, potentially reflecting the interplay of biological, structural, and policy-related factors. Tracing these patterns across four pandemic waves highlights the fluidity of inequality and the shifting relevance of different social intersections.

Our findings demonstrate that inequalities in severe COVID-19 outcomes cannot be adequately understood through one-dimensional analyses. Broad categories often treated as homogeneous—such as low income, limited education, or migration status—conceal substantial internal heterogeneity that varies across social intersections and over time. This reflects both the intersections of multiple social positions and the shifting nature of vulnerability across pandemic phases. *Intersectionality* thus serves not merely as a statistical lens, but as a theoretical framework for understanding how structural conditions and individual agency interact to produce health inequalities. These results also invite critical reflection on whose realities are captured by recommendations and interventions, and on who is considered ‘at risk’, when, and why. They reinforce the idea that intersectionality is not only a theoretical or methodological approach but also has practical implications for how social justice and health equity are either advanced or undermined in public health policy and practice (Bowleg, 2025).

Future research and policy must move beyond one-dimensional categorical models by combining theoretical insights with empirically grounded, context-specific, and adaptive approaches. Reducing health inequalities during pandemics requires more than equity-oriented crisis measures. Sustainable progress depends on long-term action to address the structural inequities embedded in everyday living conditions and broader social arrangements.

## CRediT authorship contribution statement

**Jesper Löve:** Writing – review & editing, Writing – original draft, Methodology, Investigation, Formal analysis, Conceptualization, Funding acquisition. **Gunilla Priebe:** Writing – review & editing, Methodology, Conceptualization, Funding acquisition. **Bo Burström:** Writing – review & editing, Methodology, Conceptualization, Funding acquisition. **Ailiana Santosa:** Writing – review & editing, Resources, Methodology, Formal analysis, Data curation. **Nawi Ng:** Writing – review & editing, Software, Methodology, Funding acquisition, Formal analysis, Data curation, Conceptualization.

## Ethics declaration

The study used de-identified register data; informed consent was not required under Swedish law. The SCIFI-PEARL dataset, which includes ongoing updates through at least the end of 2025, has been approved by the Swedish Ethical Review Authority (2020-01800 with subsequent amendments).

## Declaration of generative AI in the manuscript preparation process

AI-assisted tools were used for language editing and clarity. All content was reviewed by the authors, who take full responsibility for the article.

## Funding

This research has been funded by the research grant from the Swedish Research Council for Health, Working Life, and Welfare (Forte, grant number: 2021-00284). SCIFI-PEARL was supported by the SciLifeLab National COVID-19 Research Program funded by the Knut and Alice Wallenberg Foundation (KAW 2021-0010/VC 2021.0018; KAW 2020.0299/VC 2022.0008) and the Swedish Research Council (2021-05045; 2021-05450), with additional support from ALF (ALFGBG-938453; 971130; 978954; 1006729), Forte–Formas (2020-02828), and Forte (2024-01711).

## Declaration of interest

The authors declare no competing interests.

## Data Availability

The individual-level register data used in this study are not publicly available under Swedish law (Public Access to Information and Secrecy Act, 2009:400; EU General Data Protection Regulation). Access to the data can be requested from the relevant data holders—Statistics Sweden and the National Board of Health and Welfare—subject to legal and ethical approvals. Aggregated results and analytic code underlying the figures and tables are available from the corresponding author upon reasonable request.
