# Supplementary material for: Intersectional inequalities in COVID-19 hospitalisation and mortality: A nationwide MAIHDA study of Swedish adults across four pandemic waves (2020–2022)
Source: SSM Popul Health. 2026 Sep 19;36:101968. doi: 10.1016/j.ssmph.2026.101968 (PMC13657095; doi:10.1016/j.ssmph.2026.101968)
Supplement: Supplementary file 1 [file mmc1.docx]

**Supplementary Tables and Figures**

**Supplementary Table 1. Number of individuals with COVID-19 hospitalisations and deaths during the four COVID-19 pandemic waves in Sweden among population aged 18+ (n=8,065,007) across age group and sociodemographic characteristics**

|  | | | **18-69 years old** | | | | | **≥70 years old** | | | | |
| --- | --- | --- | --- | --- | --- | --- | --- | --- | --- | --- | --- | --- |
|  |  |  | **Wave 1**  **(n=6,463,844)** | **Wave 2**  **(n=6,459,200)** | **Wave 3**  **(n=6,480,304)** | **Wave 4**  **(n=6,493,544)** | **Total**  **(n=6,474,223)** | **Wave 1**  **(n=1,580,971)** | **Wave 2**  **(n=1,568,008)** | **Wave 3**  **(n=1,583,855)** | **Wave 4**  **(n=1,630,302)** | **Total**  **(n=1590,784)** |
| **Gender** | Women | Total population | 3,180,592 | 3,178,254 | 3,187,361 | 3,192,960 | 3,184,792 | 856,743 | 849,661 | 857,659 | 881,217 | 861,320 |
|  |  | Hospitalisations | 3,489 | 3,437 | 4,734 | 2,663 | 14,323 | 3,371 | 4,961 | 2,523 | 3,116 | 13,971 |
|  |  | Deaths | 146 | 107 | 143 | 98 | 494 | 2,476 | 2,496 | 707 | 837 | 6,516 |
|  | Men | Total population | 3,283,252 | 3,280,946 | 3,292,943 | 3,300,584 | 3,289,431 | 724,228 | 718,347 | 726,196 | 749,085 | 729,464 |
|  |  | Hospitalisations | 5,725 | 5,607 | 7,687 | 3,294 | 22,313 | 3,948 | 5,949 | 3,348 | 4,145 | 17,390 |
|  |  | Deaths | 441 | 252 | 374 | 187 | 1,254 | 2,699 | 2,726 | 1,085 | 1,121 | 7,631 |
| **Birthplace income classification** | Swedish | Total population | 4,980,943 | 4,975,642 | 4,968,447 | 4,973,152 | 4,974,546 | 1,396,666 | 1,383,996 | 1,397,176 | 1,435,712 | 1,403,388 |
|  |  | Hospitalisations | 4,626 | 4,831 | 7,527 | 3,206 | 20,190 | 5,775 | 8,814 | 4,610 | 5,797 | 24,996 |
|  |  | Deaths | 335 | 255 | 331 | 175 | 1,096 | 4,302 | 4,424 | 1,463 | 1,608 | 11,797 |
|  | HIC | Total population | 478,664 | 476,016 | 476,931 | 474,498 | 476,527 | 147,535 | 146,299 | 147,335 | 151,631 | 148,200 |
|  |  | Hospitalisations | 996 | 1,021 | 1,258 | 756 | 4,031 | 942 | 1,416 | 797 | 1,032 | 4,187 |
|  |  | Deaths | 64 | 33 | 63 | 39 | 199 | 652 | 630 | 223 | 252 | 1,757 |
|  | UMIC | Total population | 459,290 | 459,632 | 466,540 | 469,604 | 463,767 | 21,456 | 21,860 | 22,757 | 24,475 | 22,637 |
|  |  | Hospitalisations | 1,747 | 1,614 | 1,796 | 1,040 | 6,197 | 355 | 387 | 265 | 269 | 1,276 |
|  |  | Deaths | 74 | 43 | 49 | 37 | 203 | 124 | 104 | 55 | 57 | 340 |
|  | LMIC | Total population | 240,961 | 241,122 | 249,391 | 251,363 | 245,709 | 10,854 | 11,175 | 11,687 | 12,921 | 11,659 |
|  |  | Hospitalisations | 754 | 734 | 874 | 366 | 2,728 | 121 | 183 | 112 | 91 | 507 |
|  |  | Deaths | 36 | 14 | 33 | 11 | 94 | 48 | 41 | 28 | 25 | 142 |
|  | LIC | Total population | 303,986 | 306,788 | 318,995 | 324,927 | 313,674 | 4,460 | 4,678 | 4,900 | 5,563 | 4,900 |
|  |  | Hospitalisations | 1,091 | 844 | 966 | 589 | 3,490 | 126 | 110 | 87 | 72 | 395 |
|  |  | Deaths | 78 | 14 | 41 | 23 | 156 | 49 | 23 | 23 | 16 | 111 |
| **Education level** | High education | Total population | 2,692,869 | 2,680,861 | 2,753,141 | 2,733,227 | 2,715,025 | 411,887 | 415,240 | 425,817 | 446,765 | 424,927 |
|  |  | Hospitalisations | 2,988 | 2,958 | 4,258 | 1,669 | 11,873 | 1,505 | 2,151 | 1,239 | 1,456 | 6,351 |
|  |  | Deaths | 144 | 79 | 114 | 61 | 398 | 928 | 822 | 312 | 329 | 2,391 |
|  | Low education | Total population | 3,770,975 | 3,778,339 | 3,727,163 | 3,760,317 | 3,759,199 | 1,169,084 | 1,152,768 | 1,158,038 | 1,183,537 | 1,165,857 |
|  |  | Hospitalisations | 6,226 | 6,086 | 8,163 | 4,288 | 24,763 | 5,814 | 8,759 | 4,632 | 5,805 | 25,010 |
|  |  | Deaths | 443 | 280 | 403 | 224 | 1,350 | 4,247 | 4,400 | 1,480 | 1,629 | 11,756 |
| **Income level** | High Income | Total population | 4,389,991 | 4,368,447 | 4,104,279 | 4,068,369 | 4,232,772 | 519,441 | 527,130 | 404,361 | 431,696 | 470,657 |
|  |  | Hospitalisations | 5,826 | 5,660 | 7,692 | 2,651 | 21,829 | 2,131 | 3,059 | 1,467 | 1,380 | 8,037 |
|  |  | Deaths | 269 | 159 | 278 | 92 | 798 | 1,323 | 1,188 | 485 | 343 | 3,339 |
|  | Low Income | Total population | 2,073,853 | 2,090,753 | 2,376,025 | 2,425,175 | 2,241,452 | 1,061,530 | 1,040,878 | 1,179,494 | 1,198,606 | 1,120,127 |
|  |  | Hospitalisations | 3,388 | 3,384 | 4,729 | 3,306 | 14,807 | 5,188 | 7,851 | 4,404 | 5,881 | 23,324 |
|  |  | Deaths | 318 | 200 | 239 | 193 | 950 | 3,852 | 4,034 | 1,307 | 1,615 | 10,808 |

| **Supplementary Table 2. Incidence of COVID-19 hospitalisations and deaths per10,000 Swedish population** | | | | | | | | |
| --- | --- | --- | --- | --- | --- | --- | --- | --- |
|  | **Incidence of COVID hospitalisation (per 10,000 population)** | | | | **Incidence of COVID deaths (per 10,000 population)** | | | |
| **18-69 years** | **Wave 1**  **(n=6,463,844)** | **Wave 2**  **(n=6,459,200)** | **Wave 3**  **(n=6,480,304)** | **Wave 4**  **(n=6,493,544)** | **Wave 1**  **(n=6,463,844)** | **Wave 2**  **(n=6,459,200)** | **Wave 3**  **(n=6,480,304)** | **Wave 4**  **(n=6,493,544)** |
| Women | 10.97 | 10.81 | 14.85 | 8.34 | 0.46 | 0.34 | 0.45 | 0.31 |
| Men | 17.44 | 17.09 | 23.34 | 9.98 | 1.34 | 0.77 | 1.14 | 0.57 |
| Sweden | 9.29 | 9.71 | 15.15 | 6.45 | 0.67 | 0.51 | 0.67 | 0.35 |
| HIC | 20.81 | 21.45 | 26.38 | 15.93 | 1.34 | 0.69 | 1.32 | 0.82 |
| UMIC | 38.04 | 35.12 | 38.5 | 22.15 | 1.61 | 0.94 | 1.05 | 0.79 |
| LMIC | 31.29 | 30.44 | 35.05 | 14.56 | 1.49 | 0.58 | 1.32 | 0.44 |
| LIC | 35.89 | 27.51 | 30.28 | 18.13 | 2.57 | 0.46 | 1.29 | 0.71 |
| High income | 13.27 | 12.96 | 18.74 | 6.52 | 0.61 | 0.36 | 0.68 | 0.23 |
| Low income | 16.34 | 16.19 | 19.9 | 13.63 | 1.53 | 0.96 | 1.01 | 0.8 |
| High education | 11.1 | 11.03 | 15.47 | 6.11 | 0.53 | 0.29 | 0.41 | 0.22 |
| Low education | 16.51 | 16.11 | 21.9 | 11.4 | 1.17 | 0.74 | 1.08 | 0.6 |
| **≥70 years** | **Wave 1**  **(n=1,580,971)** | **Wave 2**  **(n=1,568,008)** | **Wave 3**  **(n=1,583,855)** | **Wave 4**  **(n=1,630,302)** | **Wave 1**  **(n=1,580,971)** | **Wave 2**  **(n=1,568,008)** | **Wave 3**  **(n=1,583,855)** | **Wave 4**  **(n=1,630,302)** |
| Women | 39.35 | 58.39 | 29.42 | 35.36 | 28.9 | 29.38 | 8.24 | 9.5 |
| Men | 54.51 | 82.82 | 46.1 | 55.33 | 37.27 | 37.95 | 14.94 | 14.96 |
| Sweden | 41.35 | 63.69 | 33 | 40.38 | 30.8 | 31.97 | 10.47 | 11.2 |
| HIC | 63.85 | 96.79 | 54.09 | 68.06 | 44.19 | 43.06 | 15.14 | 16.62 |
| UMIC | 165.45 | 177.04 | 116.45 | 109.91 | 57.79 | 47.58 | 24.17 | 23.29 |
| LMIC | 111.48 | 163.76 | 95.83 | 70.43 | 44.22 | 36.69 | 23.96 | 19.35 |
| LIC | 282.51 | 235.14 | 177.55 | 129.43 | 109.87 | 49.17 | 46.94 | 28.76 |
| High income | 41.02 | 58.03 | 36.28 | 31.97 | 25.47 | 22.54 | 11.99 | 7.95 |
| Low income | 48.87 | 75.43 | 37.34 | 49.07 | 36.29 | 38.76 | 11.08 | 13.47 |
| High education | 36.54 | 51.8 | 29.1 | 32.59 | 22.53 | 19.8 | 7.33 | 7.36 |
| Low education | 49.73 | 75.98 | 40 | 49.05 | 36.33 | 38.17 | 12.78 | 13.76 |

**Supplementary Table 3. Intraclass correlation (ICC) of different models in the multilevel analysis of COVID-19**

**hospitalisations and deaths among Swedish population.**

|  | **Intraclass correlation for COVID hospitalisation (95% CI)** | | | | **Intraclass correlation for COVID deaths (95% CI)** | | | |
| --- | --- | --- | --- | --- | --- | --- | --- | --- |
|  | **Wave 1** | **Wave 2** | **Wave 3** | **Wave 4** | **Wave 1** | **Wave 2** | **Wave 3** | **Wave 4** |
| **18-69 years** | | | | | | | | |
| Null model | 0.13  (0.08;0.2) | 0.11  (0.07;0.16) | 0.07  (0.05;0.12) | 0.1  (0.06;0.15) | 0.25  (0.15;0.36) | 0.17  (0.09;0.28) | 0.15  (0.09;0.24) | 0.22  (0.13;0.35) |
| Sex | 0.12  (0.07;0.17) | 0.1  (0.06;0.15) | 0.06  (0.04;0.1) | 0.1  (0.06;0.15) | 0.16  (0.09;0.27) | 0.14  (0.07;0.24) | 0.11  (0.06;0.17) | 0.2  (0.11;0.33) |
| Birthplace* | 0.06  (0.03;0.09) | 0.05  (0.03;0.08) | 0.04  (0.02;0.06) | 0.05  (0.03;0.08) | 0.23  (0.14;0.36) | 0.19  (0.1;0.3) | 0.14  (0.07;0.24) | 0.22  (0.11;0.37) |
| Income | 0.13  (0.09;0.19) | 0.11  (0.07;0.16) | 0.08  (0.05;0.11) | 0.09  (0.06;0.13) | 0.24  (0.15;0.37) | 0.14  (0.07;0.23) | 0.15  (0.08;0.24) | 0.15  (0.07;0.27) |
| Education | 0.12  (0.08;0.17) | 0.1  (0.07;0.15) | 0.07  (0.04;0.1) | 0.09  (0.06;0.13) | 0.23  (0.14;0.34) | 0.14  (0.07;0.25) | 0.1  (0.05;0.18) | 0.2  (0.1;0.33) |
| All variables | 0.01  (0.01;0.03) | 0.02  (0.01;0.03) | 0.01  (0.01;0.03) | 0.01  (0.01;0.03) | 0.06  (0.02;0.13) | 0.01  (0;0.05) | 0.01  (0;0.03) | 0.02  (0;0.09) |
| **≥70 years** | | | | | | | | |
| Null model | 0.14  (0.09;0.22) | 0.11  (0.06;0.17) | 0.12  (0.08;0.18) | 0.09  (0.05;0.14) | 0.09  (0.05;0.15) | 0.06  (0.04;0.11) | 0.14  (0.08;0.23) | 0.1  (0.05;0.17) |
| Sex | 0.13  (0.08;0.19) | 0.08  (0.05;0.13) | 0.1  (0.06;0.16) | 0.08  (0.05;0.13) | 0.07  (0.04;0.11) | 0.05  (0.03;0.09) | 0.09  (0.05;0.15) | 0.07  (0.04;0.13) |
| Birthplace* | 0.04  (0.02;0.07) | 0.05  (0.03;0.1) | 0.03  (0.02;0.06) | 0.04  (0.02;0.08) | 0.06  (0.03;0.11) | 0.06  (0.03;0.11) | 0.09  (0.04;0.16) | 0.06  (0.03;0.11) |
| Income | 0.14  (0.08;0.22) | 0.1  (0.06;0.16) | 0.12  (0.07;0.19) | 0.06  (0.04;0.1) | 0.07  (0.04;0.13) | 0.05  (0.03;0.1) | 0.14  (0.08;0.22) | 0.07  (0.04;0.13) |
| Education | 0.13  (0.08;0.21) | 0.1  (0.06;0.15) | 0.11  (0.07;0.17) | 0.09  (0.05;0.14) | 0.07  (0.03;0.12) | 0.04  (0.02;0.08) | 0.11  (0.06;0.19) | 0.08  (0.04;0.14) |
| All variables | 0.01  (0;0.02) | 0  (0;0.01) | 0  (0;0.01) | 0  (0;0) | 0.01  (0;0.02) | 0  (0;0.01) | 0.01  (0;0.03) | 0  (0;0.02) |

*Birthplace Income Classification

Supplement Figure 1. Interaction effect of predicted proportion

of COVID-19 hospitalisations and deaths in Sweden.
